# Cytotoxicity of portoamides in human cancer cells and analysis of the molecular mechanisms of action

**DOI:** 10.1371/journal.pone.0188817

**Published:** 2017-12-07

**Authors:** Tiago Ribeiro, Filipa Lemos, Marco Preto, Joana Azevedo, Maria Lígia Sousa, Pedro N. Leão, Alexandre Campos, Stig Linder, Rui Vitorino, Vitor Vasconcelos, Ralph Urbatzka

**Affiliations:** 1 CIIMAR, Interdisciplinary Center of Marine and Environmental Research, Porto, Portugal; 2 Department of Oncology-Pathology, Karolinska Institute, Stockholm, Sweden; 3 Department of Medical and Health Sciences, Faculty of Health Sciences, Linköping University, Linköping, Sweden; 4 Department of Medical Sciences, Institute of Biomedicine–iBiMED, University of Aveiro, Aveiro, Portugal; 5 Department of Physiology and Cardiothoracic Surgery, Faculty of Medicine, University of Porto, Porto, Portugal; 6 FCUP, Faculty of Science, Department of Biology, University of Porto, Porto, Portugal; Columbia University, UNITED STATES

## Abstract

Portoamides are cyclic peptides produced and released by the cyanobacterial strain *Phormidium sp*. presumably to interfere with other organisms in their ecosystems ("allelopathy"). Portoamides were previously demonstrated to have an antiproliferative effect on human lung carcinoma cells, but the underlying mechanism of this activity has not been described. In the present work, the effects of portoamides on proliferation were examined in eight human cancer cell lines and two non-carcinogenic cell lines, and major differences in sensitivities were observed. To generate hypotheses with regard to molecular mechanisms of action, quantitative proteomics using 2D gel electrophoresis and MALDI-TOF/TOF were performed on the colon carcinoma cell line HT-29. The expression of proteins involved in energy metabolism (mitochondrial respiratory chain and pentose phosphate pathway) was found to be affected. The hypothesis of altered energy metabolism was tested in further experiments. Exposure to portoamides resulted in reduced cellular ATP content, likely due to decreased mitochondrial energy production. Mitochondrial hyperpolarization and reduced mitochondrial reductive capacity was observed in treated cells. Furthermore, alterations in the expression of peroxiredoxins (PRDX4, PRDX6) and components of proteasome subunits (PSB4, PSA6) were observed in portoamide-treated cells, but these alterations were not associated with detectable increases in oxidative stress. We conclude that the cytotoxic activity of portoamides is associated with disturbance of energy metabolism, and alterations in mitochondrial structure and function.

## Introduction

Natural compounds have been an important source of new drugs for the treatment of many diseases [1]. Cyanobacteria are known to produce a plentitude of secondary metabolites, whose potential applications range from industrial to biomedical. For example, poly-β-hydroxybutyrate is used for the production of bioplastics [2] and compounds like the veraguamides have revealed cytotoxicity against human cancer cell lines [3]. The most famous example of a cyanobacterial anticancer drug is brentuximab vedotin, a synthetic compound with its origin from a cyanobacterial metabolite, which passed the U.S. FDA (US Food and Drug Administration) approval for clinical use. Brentuximab vedotin targets CD30 and microtubules, and is used for the treatment of anaplastic large T-cell systemic malignant lymphoma and Hodgkin’s disease [4]. Our own group demonstrated that picocyanobacteria have the capacity to induce cytotoxicity in human cancer cells via different modes of actions [5] and that hierridin B, a metabolite from the marine cyanobacterium *Cyanobium sp*. decreased mitochondrial activity and function [6].

Portoamides were previously isolated from the cyanobacteria *Phormidium* sp. LEGE 05292 from the Blue Biotechnology and Ecotoxicology Culture Collection (LEGE CC, http://www.ciimar.up.pt/legecc/), due to its allelopathic effect upon the alga *Chlorella vulgaris* [7]. Portoamides A and B individually reduced the viability of the non-small lung carcinoma cell line H460, and interestingly, stronger cytotoxicity was observed for the mixture of the two compounds [7].

In this work, the cyanobacterial strain *Phormidium sp*. LEGE 05292 was grown under standard conditions in order to isolate portoamides A and B (designated portoamides in the following sections). The aim of the study was to expand the analysis of cytotoxicity of portoamides to eight human carcinogenic and two non-carcinogenic cell lines using the MTT assay. Following these results, the most sensitive cell line was selected for analyses of the molecular mechanisms underlying the observed cytotoxicity. Proteomics was applied as a non-targeted approach using 2D gel electrophoresis and protein identification by MALDI-TOF/TOF to gain insights into altered cellular pathways. In order to complement the proteomics data, a quantitative analysis of fluorescently labeled nuclei, cytoplasm and mitochondria was performed using the CellProfiler [8] software to detect cellular alterations and phenotypic anchors. Hypotheses generated by proteomics were further tested by functional assays for oxidative stress, ROS production, redox potential, ATP level and mitochondrial (Glu/Gal) toxicity assessment.

## Results and discussion

### Isolation and purification of portoamides

*Phormidium sp*. LEGE 05292 was grown for eight months, and the total lyophilized biomass obtained was 5.35 g. Portoamides A and B (30.0 mg) were isolated by column chromatography followed by HPLC-PDA, and their presence confirmed by LC/MS with a relative proportion of A to B of 3:1 based on the PDA spectrum (S1 Fig). This naturally occurring and defined mixture of both portoamides was used for exposure of cells and called portoamides throughout the document.

### Effect of portoamides on the proliferation of different cell lines

The effects of portoamides (78 ng/mL—10 μg/mL) on the proliferation of eight human carcinogenic and two non-carcinogenic cell lines were examined using the MTT assay. Portoamides were dissolved in 0.5% dimethyl sulfoxide (DMSO), a solvent concentration that did not affect the proliferation of any of the cell lines tested. Portoamides did not affect the viability of lung carcinoma (A549), hepatocellular carcinoma (HepG2), breast ductal carcinoma (T-47D) and neuroblastoma (SHSY-5Y) cells at the concentrations tested. In contrast, portoamides showed antiproliferative effects in the cell lines of colon carcinoma (RKO, HCT116), colon-rectal adenocarcinoma (HT-29) and osteosarcoma (MG-63), and the non-carcinogenic cell lines of human brain capillary endothelial cells (hCMEC/D3) and keratinocytes (HaCa) (Table 1). HT-29 was the most sensitive cell line (IC_50_: 1.5 μg/mL) and chosen for further experiments. The exposure concentrations of 0.5 μg/mL (IC_15_) and 1.0 μg/mL (IC_25_) were selected to analyze the HT-29 cells at the phase of initial loss of viability.

**Table 1:** Viability assays for eight human cancer cell lines, and two non-carcinogenic cell lines. Viability assays were performed for eight human cancer cell lines, and two non-carcinogenic cell lines treated for 48 h with portoamides. IC_50_ values are derived from triplicates per plate, and from at least two independent assays. Portoamides did not affect the viability of lung carcinoma (A549), hepatocellular carcinoma (HepG2), breast ductal carcinoma (T-47D) and neuroblastoma (SHSY-5Y) cells at the concentrations tested.

Cell Lines	IC_50_ (μg/mL) Mean ± SD	IC_50_ (μmol/L)
**MG-63**	9.2 ± 8.3	6.03
**HCT116**	5.2 ± 1.2	3.38
**RKO**	4.6 ± 3.8	3.02
**HT-29**	1.5 ± 1.3	0.98
**hCMEC/D3**	5.5 ± 0.5	3.61
**HaCaT**	4.3 ± 1.1	2.82

Membrane-active agents and other generally toxic compounds are not expected to show variations in IC_50_ between different cell lines. Variations in sensitivities in the NCI_60_ cell line panel to different drugs has been used as an indicator for selection of drugs for further testing [9]. The difference in sensitivity to portoamides between the tested cell lines (cytotoxic, non-cytotoxic) indicated the potential to induce cytotoxicity via specific molecular targets. The non-carcinogenic cell lines (hCMEC/D3, HaCaT) are characterized by infinite cell division. HaCaT keratinocytes are described as clonogenic, but not tumorigenic [10], and have mutations in the p53 gene [11]. Future work should test portoamides on human primary cells or stem cells in order to analyze their potential toxicity on normal cells.

Potential reasons for selective cytotoxicity of portoamides in different cell lines were analyzed with the help of available scientific databases, which contain genomic and proteomic data regarding the expression of many genes/proteins in different human cell lines or primary human tissues, as well as sequence variants for genes/proteins. By focusing on the two strongest induced proteins in our data set (NDUFS3 and TALDO1), a search was performed on the Human Protein Atlas (http://www.proteinatlas.org/), the NCI-60 Proteome Database (http://129.187.44.58:7070/NCI60/main/index), and the Colorectal Cancer Atlas (http://www.colonatlas.org/). However, on the basis of available data, no differences could be observed that would argue for different cell selectivity. Future works should focus on a detailed target deconvolution, which would enable a comparison between the different selectivity in human cell lines.

### Evaluation of proteomic alteration after exposure to portoamides

**Fig 1:**
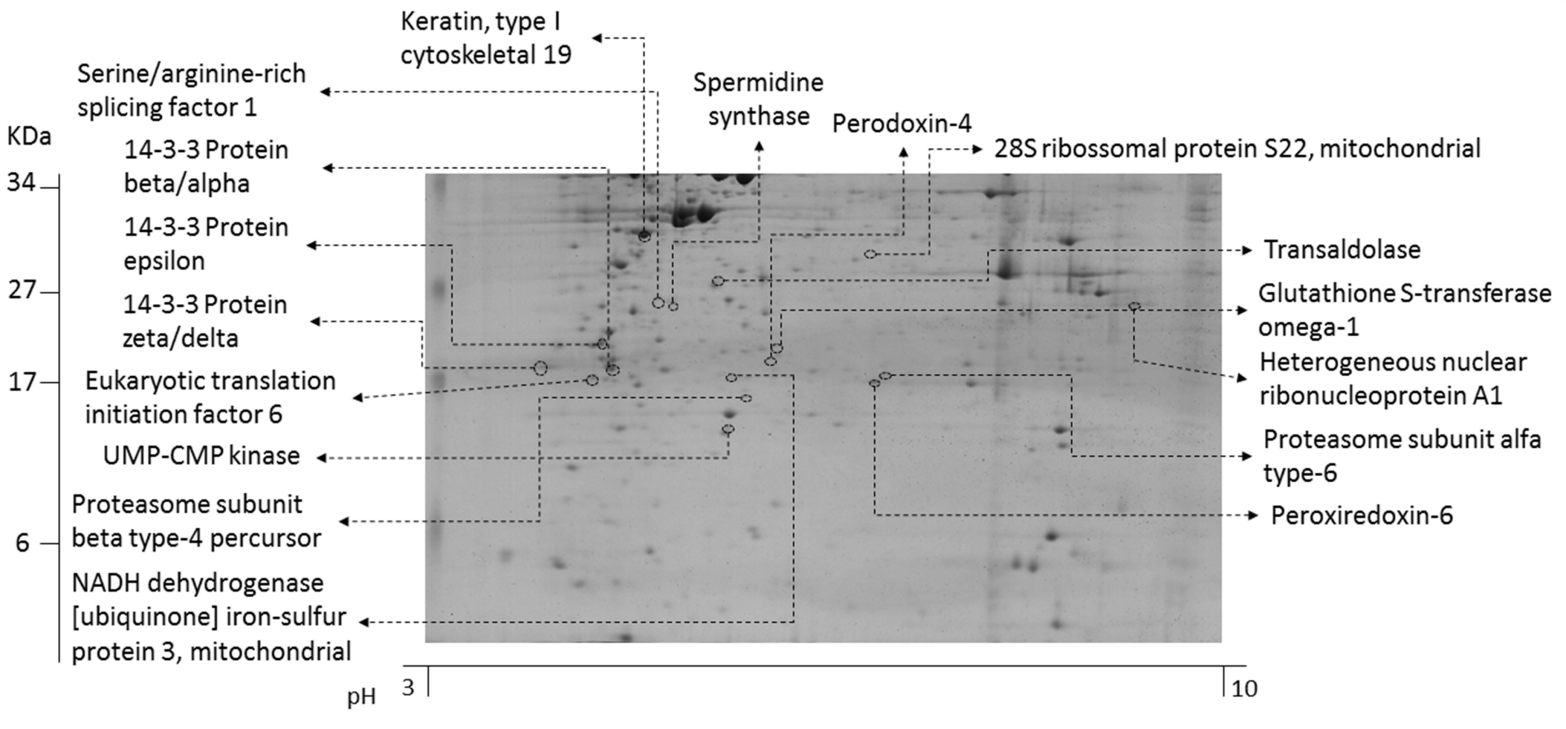
Polyacrylamide gel (12.5%) with indication of the position of the proteins identified by MALDI-TOF/TOF from the HT-29 cell line.

Drugs generally induce phenotypic changes that are useful indicators of their mechanisms of action [12]. We examined here the effects of portoamides on the proteome of HT-29 cells using 2D gel electrophoresis and mass spectrometry. The expression of 30 protein spots was observed to differ between control and treated cells. Seventeen proteins could be identified (57%), presented in Table 2, and were up-regulated. Their relative position on polyacrylamide gel is shown in Fig 1.

**Table 2:** Identification of differentially expressed spots from HT-29 cells exposed to portoamides by MALDI-TOF/TOF. The Table 2 indicates the spot number (SPP), protein name, mean intensity and standard deviation (SD), the protein accession number, protein score and the matched peptides obtained by MS and by MS/MS, of the different proteins spots of the solvent control and portoamides treatment.

SPP	Protein Name	0.5% DMSO (mean ± SD)	0.5 μg/mL portoamides (mean ± SD)	Fold (x)	1.0 μg/mL portoamides (mean ± SD)	Fold (x)	Acession Number	Gene	Protein Score	Matched Peptides	
										MS	MS/MS
**304**	Eukaryotic translation initiation factor 6	1705.4 ± 508.7	2774.4 ± 1314.2	1.6	3176.3 ± 717.7	1.9	IF6_HUMAN	EIF6	232	6	3
**1323**	14-3-3 protein zeta/delta	224.6 ± 161.8	1427.2 ± 263.0	6.4	451.1 ± 395.7	2.0	1433Z_HUMAN	YWHAZ	171	6	3
**1305**	14-3-3 protein beta/alpha	4887.4 ± 1804.0	7209.3 ± 3226.9	1.5	9801.3 ± 1574.4	2.0	1433B_HUMAN	YWHAB	228	15	3
**1401**	14-3-3 protein epsilon	4373.6 ± 1582.3	6207.2 ± 1851.9	1.4	6381.5 ± 627.7	1.5	1433E_HUMAN	YWHAE	310	16	4
**2503**	Serine/arginine-rich splicing factor 1	—	—		615.1 ± 333.3		SRSF1_HUMAN	SRSF1	57	7	2
**2902**	Keratin. type I cytoskeletal 19	—	11439.0 ± 1326.8		10723.1 ± 4165.0		K1C19_HUMAN	KRT19	128	10	1
**3408**	UMP-CMP kinase	210.5 ± 130.0	715.5 ± 117.0	3.4	—		KCY_HUMAN	CMPK1	112	11	3
**3719**	Spermidine synthase		—		106.9 ± 83.5		SPEE_HUMAN	SRM	73	8	1
**4308**	Proteasome subunit beta type-4 precursor	18.9 ± 13.0	123.7 ± 59.8	6.5	98.9 ± 76.7	5.2	PSB4_HUMAN	PSMB4	96	5	1
**4349**	Peroxiredoxin-4	25.9 ± 3.1	202.5 ± 166.3	7.8	196.9 ± 94.2	7.6	PRDX4_HUMAN	PRDX4	103	6	2
**4509**	NADH dehydrogenase [ubiquinone] iron-sulfur protein 3. mitochondrial	4.6 ± 7.0	49.1 ± 44.1	10.7	43.2 ± 41.4	9.4	NDUS3_HUMAN	NDUFS3	79	9	1
**4616**	Glutathione S-transferase omega-1	30.6 ± 12.9	40.3 ± 31.1	1.3	82.5 ± 55.4	2.7	GSTO1_HUMAN	GSTO1	64	7	1
**4709**	Transaldolase	31.5 ± 22.6	519.3 ± 232.7	16.5	944.2 ± 932.8	30.0	TALDO_HUMAN	TALDO1	153	11	2
**5302**	Peroxiredoxin-6	2146.3 ± 498.9	3147.3 ± 635.3	1.5	2975.9 ± 405.5	1.4	PRDX6_HUMAN	PRDX6	135	7	2
**5303**	Proteasome subunit alfa type-6	—	437.8 ± 395.0		—		PSA6_HUMAN	PSMA6	168	7	3
**5813**	28S ribossomal protein S22. mitochondrial	125.9 ± 57.7	—		239.5 ± 75.3	1.9	RT22_HUMAN	MRPS22	49	8	1
**9707**	Heterogeneous nuclear ribonucleoprotein A1	—	590.7 ± 178.8		301.4 ± 0.0		ROA1_HUMAN	HNRNPA1	80	14	-

The quantitative proteomic analyses pointed to interesting alterations in the expression of cytoplasmic and mitochondrial proteins, and on signaling processes. The proteins with the highest rate of alterations were involved in the mitochondrial respiratory chain (NADH dehydrogenase from the mitochondrial complex I, NDUFS3) and in the pentose phosphate pathway (PPP) (transaldolase, TALDO). The strong increase of NDUFS3 at both exposure concentrations revealed alterations of mitochondrial metabolism and energy production. Higher activity of NDSUF3 accompanied by a rise in the production of ROS is one of several causes of cellular oxidative stress, which can induce apoptosis [13], also known as ROS dependent apoptosis. Compounds such as retinoic acid induced the activity of the mitochondrial respiratory chain via NDUFS3, which led to cytotoxicity in cancer cells, overproduction of ROS and loss of mitochondrial function [14]. TALDO is the rate-limiting enzyme of the non-oxidative branch of PPP, which ensures the formation of ribose-5-phosphate for nucleotide (DNA, RNA) biosynthesis and of NADPH for neutralization of ROS [15]. TALDO deficiency in mice led to the depletion of NADPH and GSH, loss of mitochondrial transmembrane potential and mitochondrial mass, and was linked to many clinical diseases such as male infertility, chronic liver diseases and hepatic carcinoma [15]. The action of TALDO varied in a cell-type specific manner, but in Jurkat cells the overexpression of TALDO accelerated NADPH turnover and increased cellular sensitivity to apoptosis induced by hydrogen peroxide or nitric oxide [16], and elevated the mitochondrial membrane potential [15]. Furthermore, two subunits of the proteasome (PSA6_HUMAN e PSB4_HUMAN) were increased in both portoamide exposure groups in comparison with the solvent control. The proteasome is a key component in the response to cellular stress, but also in the regulation of the cell cycle and of apoptosis. Two peroxiredoxins were increased, peroxiredoxin-4 (PRDX4) and peroxiredoxin-6 (PRDX6), two important anti-oxidant enzymes and scavengers of ROS, as well as GST1, an enzyme responsible for protein modification related with cellular redox variations [17]. Summarizing the proteomics results, two main hypotheses can be generated that were tested in the following experiments; (1) portoamides induce oxidative stress that may lead to ROS induced apoptosis, (2) portoamides affect energy metabolism and in particular mitochondrial activity/function.

### Portoamides do not induce oxidative stress

**Fig 2:**
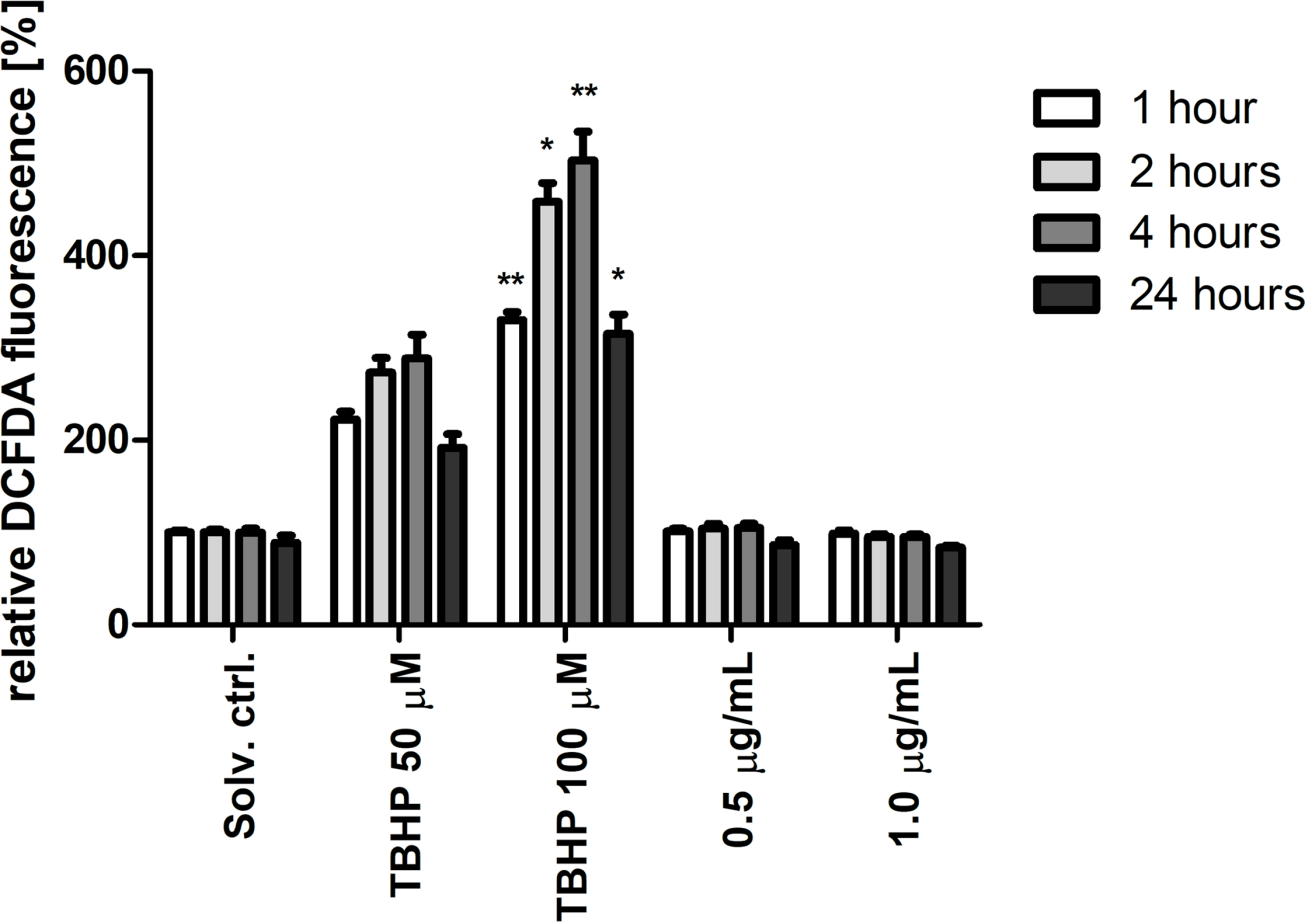
Detection of cellular ROS production using DCFDA reagent after exposure to portoamides (0.5 μg/mL and 1.0 μg/mL) and positive controls (tert-Butyl hydroperoxide, TBHP at 50 μM and 100 μM). Values are shown relative to the respective solvent control (DMSO 0.5%) of each time period (1, 2, 4, 24 hours). Statistically significant differences between the respective solvent control and the treatment groups are indicated by asterisks (Kruskal-Wallies, Dunn’s test, * = p<0.05, ** = p<0.01).

**Fig 3:**
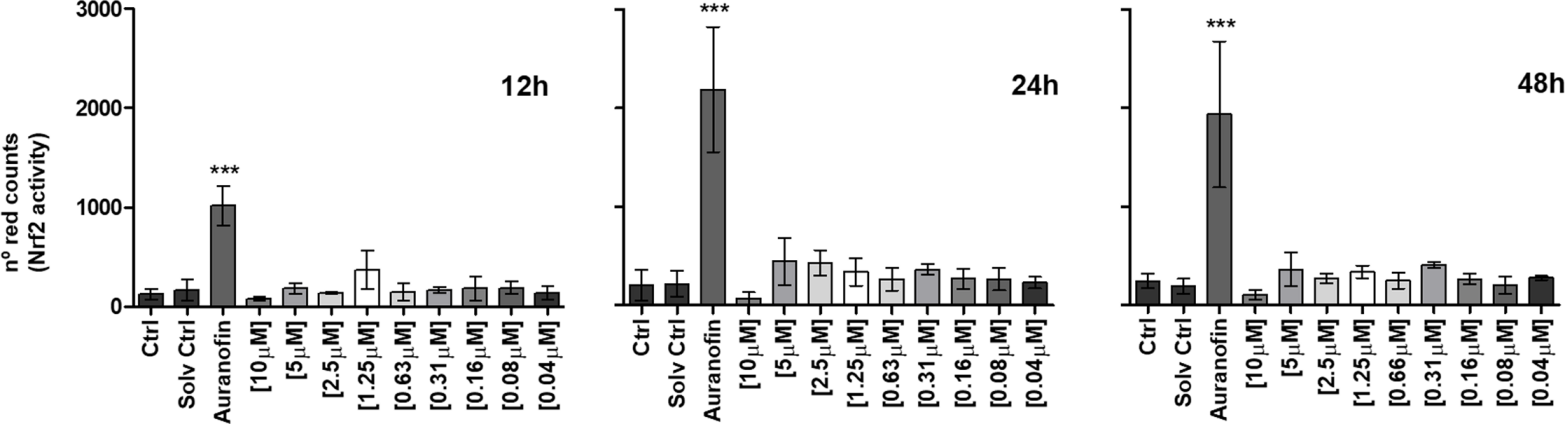
Nrf2 activity in HCT116 cells transfected with the plasmid pTRAF. HCT116 cells were cultured at different concentrations of portoamides, the solvent control was 0.3% DMSO, and fluorescent signals were analyzed with the Incucyte ZOOM software. Statistically significant differences between the solvent control and the treatment groups are indicated by asterisks (One-Way ANOVA, Dunnett’s test, *** = p<0.001.

The increases in TALDO, NDUFS3, GSTO1, PRDX4 and PRDX6 were all consistent with the generation of oxidative stress in exposed cells. We therefore examined ROS levels in portoamide-treated HT-29 cells using the redox-sensitive probe DCFDA. DCFDA fluorescence increased after treatment with tert-Butyl hydroperoxide (TBHP) but did not, however, increase in portoamide exposed cells (Fig 2). We also used a reporter gene assay based on the antioxidant/electrophile response element (ARE/EpRE) known to be stimulated by the transcription factor Nrf2. Nrf2 is stabilized and translocated to the nucleus under condition of oxidative stress. The reporter has previously been demonstrated to respond to conditions of oxidative stress [18]. Whereas the thioredoxin reductase inhibitor auranofin induced strong increases in Nrf-2 reporter activity, no effect was observed using portoamides (Fig 3). These findings do not support the concept that portoamides induce oxidative stress.

### Decreased mitochondrial reductive activity in portoamide-treated cells

**Fig 4:**
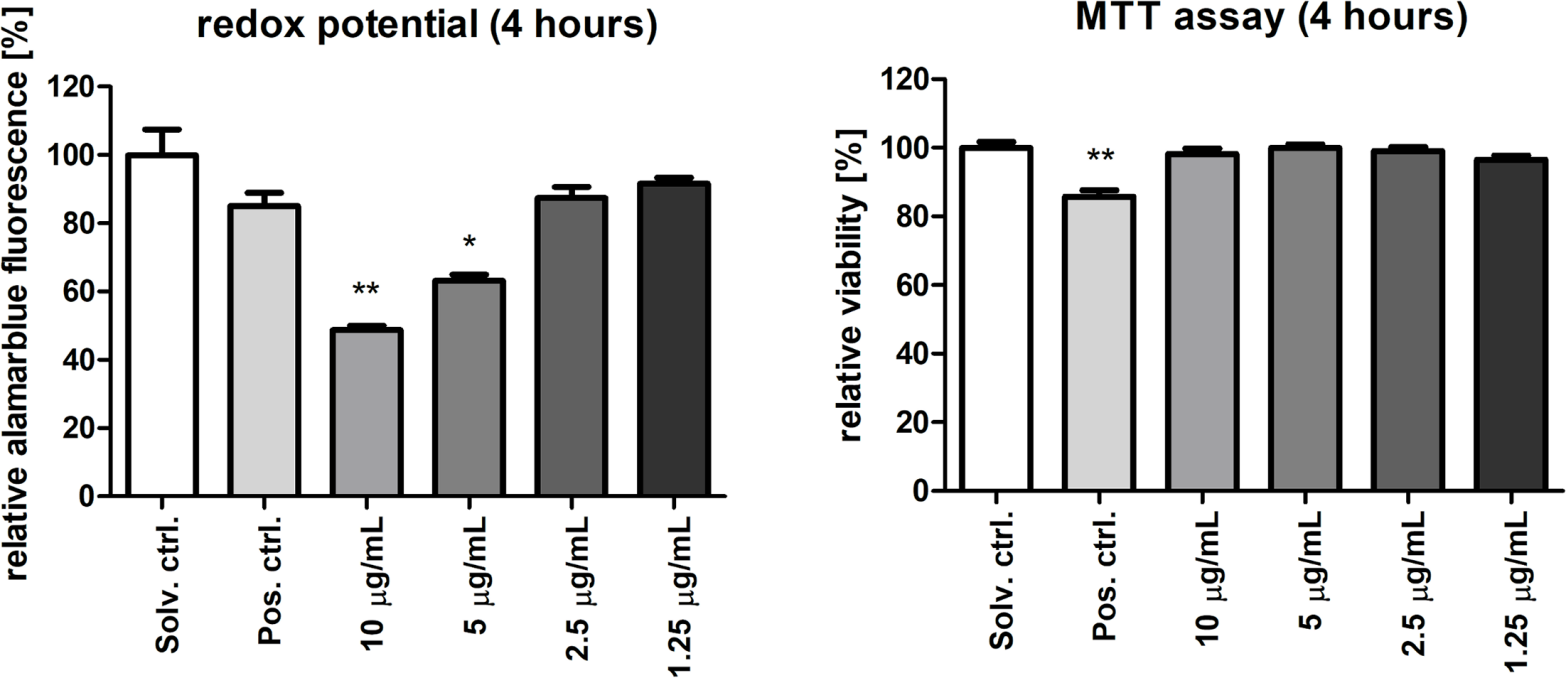
Evaluation of cellular redox status using the alamar blue assay (left panel) and viability by MTT (right panel). Values are shown relative to solvent control (DMSO 0.5%) after exposure to portoamides for 4 hours. Statistically significant differences between the solvent control and the treatment groups are indicated by asterisks (Kruskal-Wallies, Dunn’s test, * = p<0.05, ** = p<0.01). DMSO 20% was used as a positive control.

The reductive capacity of mitochondria can be assessed by determining reduction of resazurin (oxidized form: 7-hydroxy-3H-phenoxazin-3-1-10-oxide) to resorufin (Alamar Blue assay) [19]. Significant decreases in reductive capacity were observed after four hours of exposure of HT-29 cells to 5 and 10 μg/mL of portoamides (Fig 4). Metabolic activity may also be determined using tetrazolium dyes (MTT assay), widely used as viability assay. Tetrazolium reduction occurs in mitochondria, but is mainly due to NAD(P)H-dependent oxidoreductases situated in the cytosolic compartment of the cell [20]. Interestingly, no alterations in tetrazolium dye reduction were observed in cells after four hours of exposure to portoamides (Fig 4), suggesting that mitochondrial reductive capacity was selectively affected, under conditions in which no cytotoxicity was present. Results indicated that the redox potential was more oxidative compared to the solvent control. More oxidative redox potentials are typical for cells undergoing apoptosis and several protein redox switches exist as for p53, HIF1α or Bax [21].

### Portoamides decreased cellular ATP levels, and were not mito-toxic

**Fig 5:**
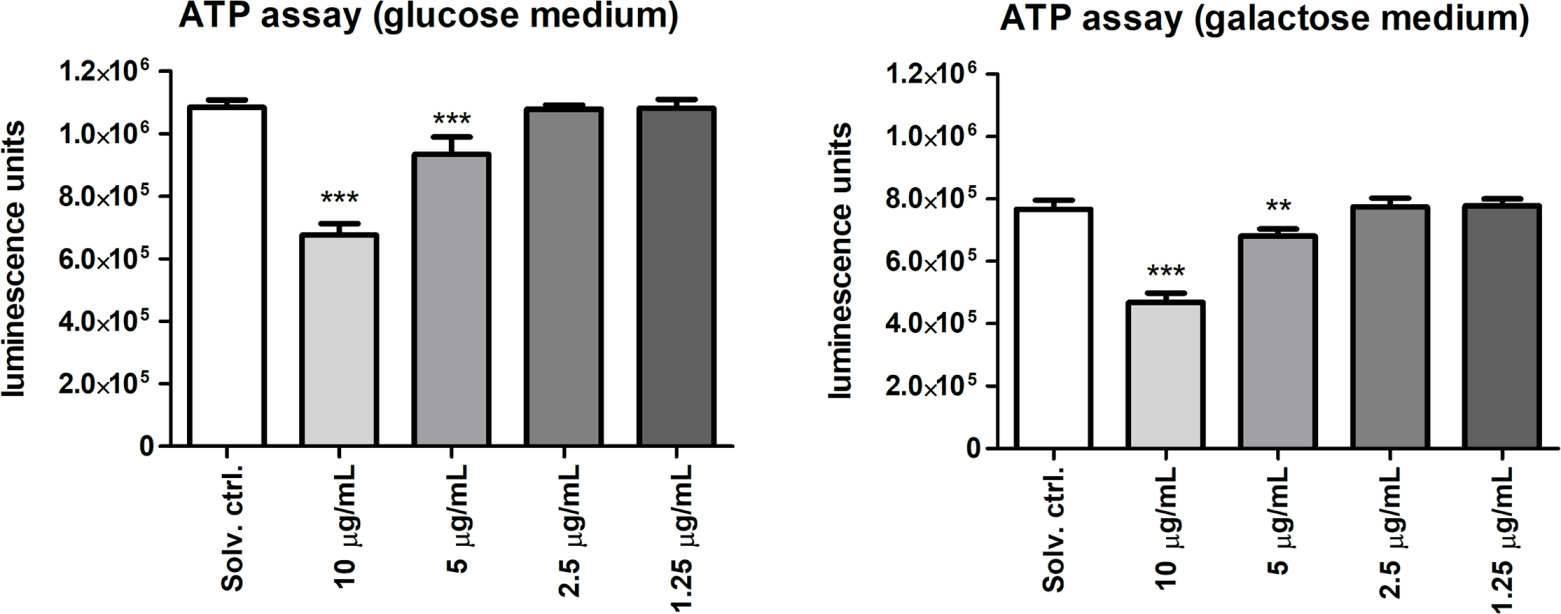
Quantification of ATP levels using selective media conditions. ATP levels were measured in HT-29 cells cultured in glucose (25 mM) and galactose (10 mM) conditioned media, which are known as a cellular switch, where cells either rely on glycolysis and oxidative phosphorylation or solely on oxidative phosphorylation, respectively. Results are expressed in luminescence units, and statistically significant differences between the solvent control and the treatment groups are indicated by asterisks (One-Way ANOVA, Dunnett’s test, ** = p<0.01, *** = p<0.001).

The proteome analysis indicated effects of portoamides on HT-29 mitochondrial metabolism. In order to provide more data on the potential alteration of mitochondrial activity, glucose or galactose conditioned media were applied, which are known as a cellular switch, where cells either rely on glycolysis and oxidative phosphorylation (OXPHOS) or solely on OXPHOS, respectively, to generate ATP, and hence energy [22,23]. In glucose media, energy can be produced via glycolysis or OXPHOS. However, since oxidation of galactose to pyruvate yields no net ATP, galactose-containing media reveal the importance of mitochondrial OXPHOS for energy production. ATP levels were significantly reduced after four hours exposure to 5 and 10 μg/mL portoamides in both glucose and galactose media (Fig 5); but a higher level of ATP was present in glucose media. These results suggest that the effect of portoamides was primarily at the level of mitochondrial energy production.

**Fig 6:**
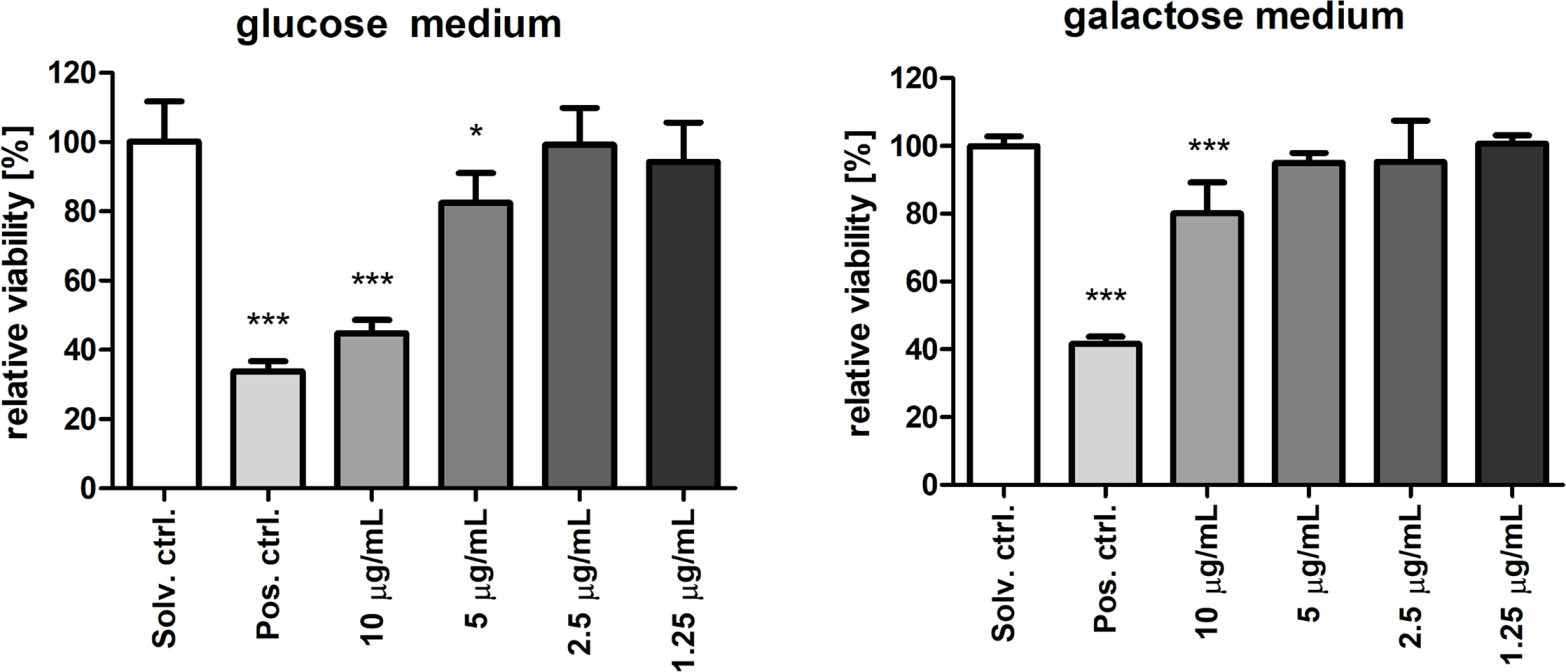
Mito-toxicity evaluation using MTT on selective media. Values are represented relative to the solvent control (DMSO 0.5%) for HT-29 cells cultured in glucose (25 mM) and galactose (10 mM) conditioned media, which are known as cellular switch, where cells either rely on glycolysis and oxidative phosphorylation or solely oxidative phosphorylation, respectively. HT-29 cells were exposed to four different portoamide concentrations, using DMSO 20% as a positive control. Statistically significant differences between the solvent control and the treatment groups are indicated by asterisks (One-Way ANOVA, Dunnett’s test, * = p<0.05, *** = p<0.001).

Next, the effects of portoamides on cell proliferation/cell viability were examined in glucose- or galactose-containing media. Mitochondrial dysfunction in response to 24 hour exposure to many different known mito-toxic compounds led to higher cytotoxicity in galactose conditioned media compared to glucose media [22]. A stronger anti-proliferative effect is expected for mito-toxic compounds in galactose-containing medium, since compensatory glycolysis cannot be activated. Portoamides reduced proliferation under both conditions (Fig 6), which demonstrated that portoamides were not mito-toxic.

### Portoamides affect mitochondrial morphology

**Fig 7:**
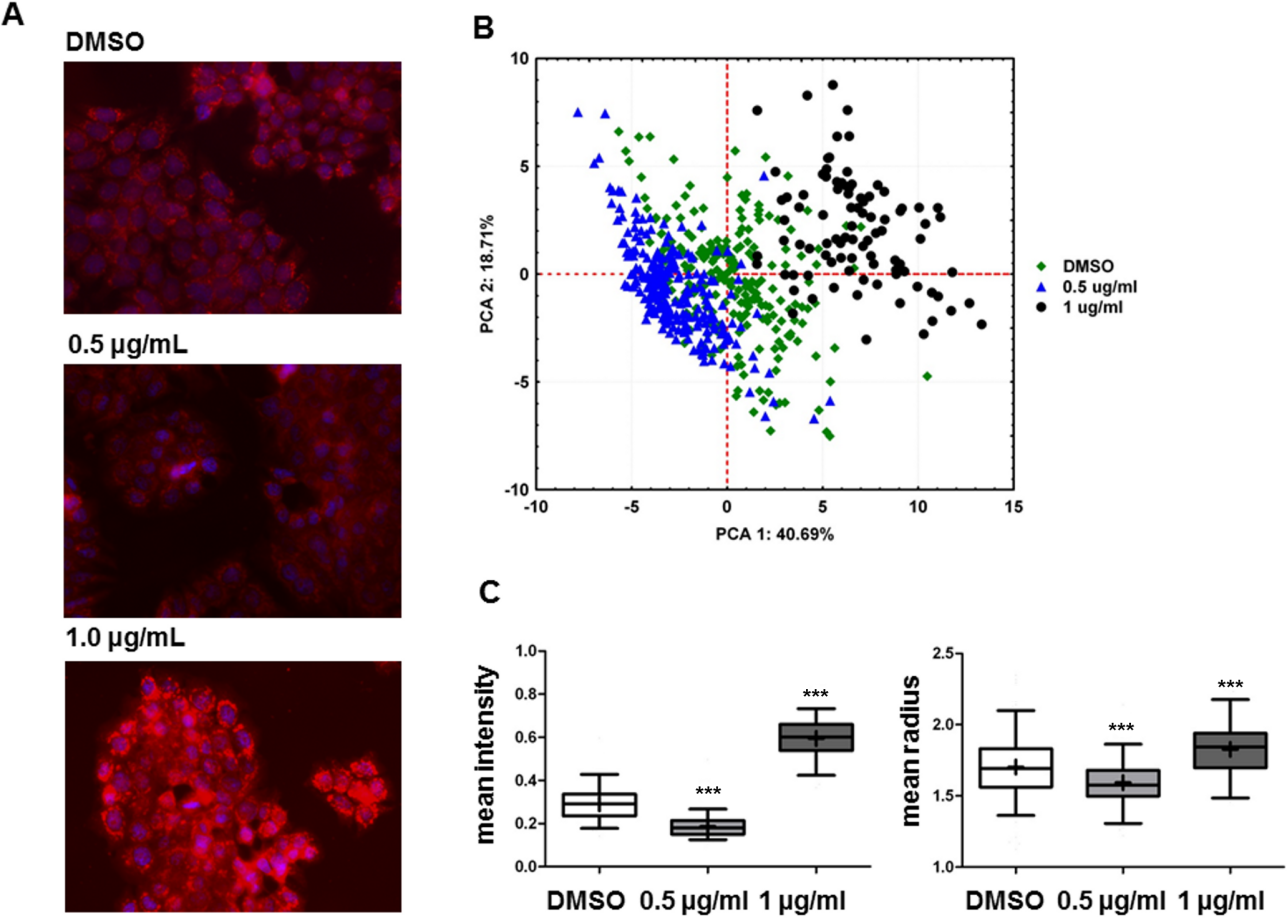
Quantitative analysis of fluorescence microscopy by CellProfiler software. A) Overlay of two fluorescent channels: blue, nucleus (HO-33342); red, mitochondria (MitoTracker). B) Principal component analysis of 40 mitochondrial parameters (object intensity, size and shape of objects) discriminated between treatments (portoamides 0.5 μg/mL, 1 μg/mL) and solvent control (DMSO). Factors that contributed mainly (> 0.85) to PC1 were related to intensity measurements (IntegratedIntensityEdge, LowerQuartileIntensity, MaxIntensityEdge, MaxIntensity, MeanIntensityEdge, MeanIntensity, MedianIntensity, MinIntensityEdge, MinIntensity, StdIntensityEdge, UpperQuartileIntensity), while parameters related to size and shape (AreaShape_Area, AreaShape_MaxFeretDiameter, AreaShape_MaximumRadius, AreaShape_MeanRadius, AreaShape_MedianRadius, AreaShape_MinFeretDiameter, AreaShape_MinorAxisLength, AreaShape_Perimeter) contributed mainly (> 0.85) to PC2. C) Detailed analysis of two mitochondrial parameters, mean fluorescence intensity and mean radius, are shown. Data are represented as box-whisker plots and statistically significant differences between the solvent control and the treatment groups are indicated by asterisks (Kruskal-Wallies, Dunn’s test, *** = p<0.001).

We next examined the effect of portoamides on mitochondria using principal component analysis of 40 different parameters recorded after staining with the membrane potential-dependent dye MitoTracker CMXRos (Fig 7, S1 Dataset). Factors that contributed mainly (> 0.85) to PC1 were related to intensity measurements, while parameters related to size and shape contributed mainly (> 0.85) to PC2. Two parameters were chosen for further statistical analysis (contributing either to PC1 or PC2), and mean fluorescence intensity and mean radius were increased in the 1.0 μg/mL portoamide treatment relative to the solvent control, while the same parameters were diminished in the 0.5 μg/mL portoamide treatment (p < 0.001). Since uptake of the MitoTracker CMXRos is dependent on the membrane potential, increased fluorescence intensity suggests increased activity or hyperpolarization of the mitochondrial membrane.

The above described increase of mitochondrial fluorescence intensity and the mean radius in the portoamide-treated cells provides support for alterations in mitochondrial activity (in line with NDUFS3). Another cyanobacterial metabolite, hierridin B, targeted mitochondrial activity in HT-29 cells, but in the opposite way to portoamides, by strongly reducing the mitochondrial fluorescence intensity and increasing level of VDAC1, protein responsible for formation of mitochondrial channels [6]. The heterodimer of 14-3-3 ε (YWHAE) and 14-3-3 ζ (YWHAZ) was originally described as a mitochondrial import stimulating factor [24] and accordingly both proteins were increased in portoamide-exposed cells, which may be related to the observed increase of mitochondrial size. The 14-3-3 protein family is described to control the mitochondria membrane permeability. High concentrations of 14-3-3γ subunits opposed the development of the mitochondria permeability transition pore (MPTP), which led to the swelling of the mitochondria [25]. However, the family of 14-3-3 proteins are keys proteins in many signaling pathways, amongst other in apoptosis and cell proliferation [26], which makes the interpretation of observed profiles difficult.

## Conclusion

We conclude that portoamides show differential toxicities in some carcinogenic and non-carcinogenic cell lines, suggesting specific mechanisms of action. Data showed that portoamides exposure in the most sensitive cell line HT-29 affected mainly mitochondrial metabolism (NDUFS3, 14-3-3 proteins, ATP level, redox potential) and various indicators of mitochondrial size and shape. Portoamides did not, however, induce oxidative stress, increase ROS or mito-toxicity. Mitochondrial bioenergetics is required for tumorigenesis [27,28], and drugs that compromize mitochondrial metabolism are expected to be particularly effective in cell populations residing in poorly vascularized areas. Poor oxygen and nutrient availability in such areas lead to limited tumor metabolic plasticity and sensitivity to inhibition of mitochondrial function [29,30].

The effects of portoamides on mitochondria were unexpected, characterized by increased mitochondrial polarization and decreased ATP production. Similar phenomena have, however, been reported in the literature [31] and attributed to the inhibition of F0F1-ATPase or adenine nucleotide transporter (ANT) activity under conditions of normal dislocation of protons from mitochondria. This will be expected to result in an elevated proton gradient and a drop in ATP levels. Finally, ROS will not necessarily be elevated under conditions of normal electron transport chain function.

**Fig 8:**
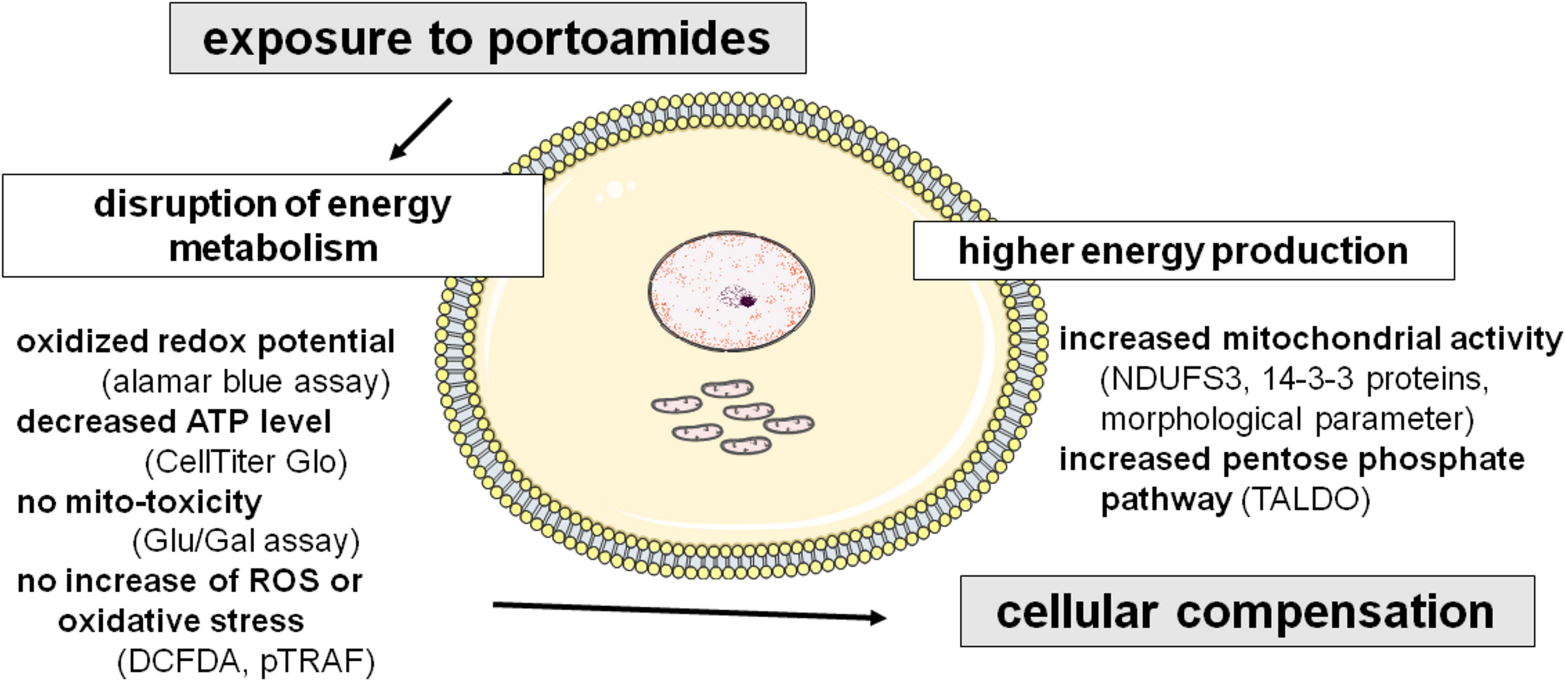
Predicted mechanism of action of portoamides in HT-29 colon adenocarcinoma cells, based on all experimental data.

Speculating on a potential mechanism (Fig 8), we propose that the energy metabolism is disrupted by portoamide exposure. Acute exposure experiments (4 hours) pointed to a more oxidative redox potential and decreased ATP level. Longer exposure time (48 hours) revealed a cellular response to generate more energy by increased mitochondrial electron transport chain (NDUFS3) and pentose phosphate pathway (TALDO).

## Experimental section

### Growth of *Phormidium sp*. LEGE 05292

*Phormidium sp*. LEGE 05292 was obtained from the Blue Biotechnology and Ecotoxicology Culture Collection (LEGE CC) and grown under standard conditions to obtain sufficient quantity of biomass as previously described [7].

### Isolation and purification of portoamides A and B

The purification of portoamides A and B was performed as previously described [7], using 5.0 g (dry mass) of *Phormidium sp*. LEGE 05292 biomass with slight alterations as detailed below. The fraction eluted at 100% methanol (MeOH) was purified and qualitatively analyzed on a HPLC (Alliance e2695) linked to a PDA 2998 detector and an automatic fraction collector III from Waters (Waters, Milford, Massachusetts, USA). The software Empower 2 (Chromatography Data Software) was used for data interpretation. The chromatographic column, XB-C18 Aeris PEPTIDE (150 mm × 4.6 mm i.d, 3.6 μm, Phenomex, Torrance, California, USA), was kept at 35°C, and the solvents were acetonitrile and ultra-pure water, both acidified with 0.1% trifluoroacetic acid (TFA) with a flux of 0.8 mL/min. The PDA ranged from 210 to 280 nm at 254 nm and a resolution of 1.2 nm. For the purification, 500 μL was injected in 0.1% MeOH, and separated by a gradient of MeOH from 50% to 100% in 35 minutes. The mixture of portoamides A and B eluted between tR = 13–15 min. For the analytic method, 20 μL were injected and separated by isocratic elution with 65% of MeOH acidified with 0.1% TFA.

### Monitoring of the portoamides A and B mixture by Liquid Chromatography–Mass Spectrometry (LC-MS)

The collected portoamides A and B mixture was lyophilized, and re-suspended in 100% acetonitrile (ACN) for LC-MS analysis. The sample was injected in the liquid phase chromatograph (Finningan, Surveyor) linked to a Mass Spectrometry detector (MS LCQ Fleet Ion Trap) equipped with an electronic ionization source, both from Thermo Finningan (Thermo Scientific, San Jose, California, USA). For data acquisition and analysis, the software Xcalibur version 2 (Thermo Scientific) was used. Nitrogen served as nebulizer gas (80) and auxiliary gas (20), both in arbitrary units. The capillary potential was 22kV and the temperature of the metallic capillary was 350°C. The spray voltage was 5.5 kV and 120V for the tubular lens. The chromatographic column used was the Hypersil GOLD (100 × 4.6 mm, 5 μL) (Thermo Scientific). The elution was done with acidified solvents with 0.1% formic acid in a flow of 0.8 mL/min with the following gradient: 40 minutes 80% H_2_O / 20% ACN; 8 minutes 100% ACN; 7 minutes 80% H_2_O / 20% ACN. The injection volume was 20 μL in a partial loop. The samples were injected in both positive and negative modes, in full scan (200–2000 m/z).

### Cell culture

The human cell lines HT-29, SH-SY5Y and T-47D were obtained from Sigma-Aldrich (St. Louis, Missouri, USA). A549, MG-63, RKO, HepG2 and HaCaT human cell lines were obtained from the American Type Culture Collection (ATCC) (Manassas, Virginia, EUA). The hCMEC/D3 cells were kindly donated by Dr. P. O. Courad (INSERM, France). All cell lines with the exception of SH-SY5Y were grown in Dulbecco Modified Eagle Medium (DMEM) from Gibco (Thermo Fisher Scientific, Waltham, Massachusetts, USA) supplemented with 10% fetal bovine serum (Biochrom, Berlin, Germany), 1% penicillin/streptomycin (Biochrom) at 100 IU/mL and 10 mg/mL, respectively, and 0.1% amphotericin (GE Healthcare, Little Chafont, United Kingdom). The cell line SH-SY5Y was grown in a 1:1 mix of the Eagle´s Minimum Essential Medium (MEM) with F12 Nutrient Mixture (HAM´s) medium, both from Life Technologies (Thermo Fisher Scientific), supplemented as described above, plus 1% of non-essential amino acids. Cells were grown in an incubator at 37°C and 5% CO2.

### Cell viability evaluation by MTT

The MTT assay (3-(4,5-dimethylthiazol-2-yl)-2,5-diphenyltetrazolium bromide) was used to assess the cytotoxicity of different concentrations of portoamides on the studied cell lines as described in [5]. Cells were seeded in 96-well plates at 1 x 10^4^ cells/cm^2^ and the final exposure concentrations of portoamides ranged from 78 ng/mL to 10μ/mL. Based on the molarity of the portoamides (A ≈ 1532 g/mol and B ≈ 1502 g/mol) and the relative proportion (3:1), a molar mass of 1525 g/mol was attributed to the portoamides, and IC_50_ values converted toμ/L (Table 1).

### Exposure of HT-29 to portoamides

For proteomics, HT-29 cells were seeded in 6-well plates at a density of 1.3 x 10^5^ cells/cm^2^ for 24 hours before experiments. Then, the medium was replaced by fresh DMEM medium with: 1) DMSO 0.5% (solvent control); 2) 0.5 μg/mL portoamides; and 3) 1 μg/mL portoamides. Six replicates per group were exposed for 48 hours. For fluorescence microscopy, HT-29 cells were seeded at a concentration of 1.3 × 10^5^ cells/cm^2^ in a 24-well plate (Orange Scientific, Braine-l'Alleud, Belgium) on a sterile glass cover slip, and three replicates were done per group.

### Protein extraction

After exposure, cells were washed two times with PBS, before 600 μL of trypsin (Life Technologies, Thermo Fischer Scientific) was added per well. Cells were transferred to 2 mL microcentrifuge tubes and centrifuged at 3000 ×g for 5 minutes. The supernatant was removed and solubilization buffer (7 M Urea, 2M Thiourea, 4% CHAPS, 65 mM Dithiothreitol (DTT) and 0.8% ampholytes (v/v), all from Sigma-Aldrich) was added (80 μL per 15 mg of cell pellet). Each tube was vortexed for two periods of 20 seconds, placed on ice for 30–45 minutes, before centrifugation at 16000 ×g for 20 minutes at 4°C. The supernatants were collected and protein content quantified with the Bradford method according to the instructions of the manufacturer (Bradford Protein Assay, Bio-Rad, Hercules, California, USA). Samples were stored at -20°C until analysis.

### Two dimensional gel electrophoresis (2DGE)

The Isoelectric focusing (IEF) of each sample was done based on [5] using immobilized pH gradient (IPG) strips (pH 3–10, 17cm) (BioRad). For SDS-PAGE electrophoresis, 12.5% polyacrylamide gels were used in cooled vertical electrophoresis unit (Hoefer, SE900, Massachusetts, USA) connected to a power supply (Hoefer, PS600). Six gels were run simultaneously with constant amperage of 480 mA for six hours. After electrophoresis, gels were fixed for 24 hours in a 200 mL solution of MeOH (40% v/v) (Merck, New Jersey, USA) and acetic acid (10% v/v) (Panreac, Barcelona, Spain) per gel. Each gel was stained for 24 hours with Coomassie Blue as described in [32]. After staining, each gel was washed with distilled water, and stored in 200 mL of ammonium sulfate (20% p/v). Images of the gels were acquired using Quantity One (BioRad) and the densitometer GS-800 (Bio-Rad), image analysis with the PDQuest 2D analysis software (BioRad) as described in [5].

### Protein identification by mass spectrometry

Differentially expressed spots were excised from the gels, washed, dehydrated, digested in trypsin (Promega, Madison, Wisconsin, USA), desalted and concentrated with 10 μl C18 reverse-phase tips (Pierce, Thermo Fisher Scientific) and spotted in duplicate on a MALDI plate, as described in [5]. Peptide mass spectra were obtained on a MALDI-TOF/TOF mass spectrometer (4800 Proteomics Analyzer, Applied Biosystems Europe) in the positive ion reflector mode. Spectra were obtained in the mass range between 800 and 4500 Da with ca. 1500 laser shots. For each sample spot, a data dependent acquisition method was created to select the six most intense peaks, excluding those from the matrix, trypsin autolysis, or acrylamide peaks, for subsequent MS/MS data acquisition. Spectra were processed and analyzed by the Global Protein Server Workstation (Applied Biosystems), which uses internal MASCOT software (v2.1.0 Matrix Science, London, UK) on searching the peptide mass fingerprints and MS/MS data. Swiss-Prot nonredundant protein sequence database (October 2014) was used for all searches under taxonomy *Homo sapiens*. Database search parameters were as follows: carbamidomethylation and propionamide of cysteine as a variable modification, oxidation of methionine, and the allowance for up to two missed tryptic cleavages. The peptide mass tolerance was 25 ppm and fragment ion mass tolerance was 0.3 Da. Positive identifications were accepted up to 95% of confidence level.

### Morphological evaluation by fluorescence microscopy

After 48 hours exposure, cells were stained with 5 μg/mL Hoechst 33342 (HO-33342) (Sigma-Aldrich), 2 μg/mL Acridine Orange (AO) (Sigma-Aldrich), 500 nM Mitotracker Red CMXRos (Life Technologies) in PBS for 10 minutes at 37°C, fixed in 4% paraformaldehyde (VWR, Radnor, Pennsylvania, USA) for 10 minutes and then mounted on microscope slides (FluoroMount, Sigma-Aldrich). Cells were observed under a fluorescence microscope Olympus BX41 (Olympus America Inc., Melville, New York, USA) with fixed excitation times for each fluorescence channel (blue, green, red; 80 ms, 25 ms, 10 ms, respectively). Selected parameters were automatically quantified by the software CellProfiler [8]. The following modules were included in the pipeline: object intensity (19 parameters), size and shape of objects (21 parameters). A complete list of parameters can be found on the manual of CellProfiler (available at http://cellprofiler.org/).

### Evaluation of oxidative stress with pTRAF transformed cells

We acknowledge the gift of the pTRAF plasmid from Elias Arnér, Division of Biochemistry, Department of Medical Biochemistry and Biophysics, Karolinska Institutet, Stockholm, Sweden. Colorectal carcinoma cell line HCT116 transformed with a pTRAF plasmid [18,33] were cultured in Dulbecco’s modified Eagle’s (DMEM) supplemented with 10% fetal bovine serum at 37°C in 5% CO_2_, 1% of penicillin/streptomycin and 0.1% of amphotericin B (Biochrom, United Kingdom). Cells were seeded in 96-well culture plates at a 1.0 x10^4^ cells/cm^2^, and adhesion allowed for 24 hours. Portoamides were tested on a concentration-response assay (up to 10 μM) and incubated on an Incucyte® ZOOM Live-Cell Analysis System (Essen Instruments, Ann Arbor, MI). For the duration of 48h, Nrf2 activity was read at λex = 585 nm, emission filter: 625–705 nm, and auranofin (6 μM) was used as a positive control to confirm Nrf2 activity [18].

### Detection of cellular reactive oxygen species (ROS)

Cells were seeded in a black-sided 96-well plate at 7.5 × 10^4^ cells/cm^2^ and allowed to attach overnight. Afterwards, cells were washed with PBS and stained with 25 μM 2’,7’–dichlorofluorescin diacetate (DFCDA) (Sigma-Aldrich) in PBS for 45 minutes in the dark at 37°C. Cells were then washed with PBS and treated with portoamides (0.5 μg/mL and 1.0 μg/mL) for 1, 2, 4 and 24 hours. DMSO at 0.5% was the solvent control and tert-butyl hydrogen peroxide (TBHP) at 50 μM and 100 μM the positive controls. Fluorescence was measured at 485 nm excitation and 535 nm emission at a Fluoroskan Ascent CF (MTX Lab Systems, Florida, USA).

### Evaluation of cellular redox status using the AlamarBlue assay

Cells were seeded in a 96-well plate at 1.8 × 10^4^ cells/cm^2^, allowed to attach overnight and exposed to a gradient of portoamide concentrations from 80 ng/mL to 10 μg/mL in 1:2 dilution steps. DMSO at 0.5% was the solvent control and DMSO at 20% was the positive control. After 1 hour, 10 μL of AlamarBlue (Thermo Fischer Scientific) was added to each well and further incubated for 3 hours (total exposure time 4h). Fluorescence was read at 530 nm excitation and 590 nm emission in a multimode microplate reader (Biotek, HT Synergy).

### Quantification of ATP levels using glucose and galactose conditioned media conditions

Cells were seeded at 3.6 × 10^4^ cells/cm^2^ in complete DMEM medium and after 24 hours changed to glucose (25 mM) or galactose (10 mM) conditioned media as described in [22]. After 6 hours pre-incubation, cells were exposed for 4 hours to four portoamide concentrations (1.25–10 μg/mL). 100 μL of CellTiter-GLo® Reagent (Promega) was added to each well, and mixed on an orbital shaker for 2 minutes. The plate was incubated at room temperature for 10 minutes in the dark and luminescence read in a multimode microplate reader (Biotek, HT Synergy) at 1 sec integration time, and gain of 125.

### Cytotoxicity evaluation using glucose and galactose selective medium conditions

Cells were seeded at 3.6 × 10^4^ cells/cm^2^ in complete DMEM medium and after 24 hours changed to glucose (25 mM) and galactose (10mM) conditioned media as previously described. After 6 hours pre-incubation, cells were exposed to a concentration gradient of portoamides from 78 ng/mL to 10 μg/mL for 24 hours. The MTT assay was then performed as described before.

### Statistical analyses

The IC_50_ values were calculated from dose-response curves of portoamides on each cell line with a nonlinear regression choosing a variable slope with four parameters. The range was defined by 0% of viability for the highest concentration of portoamides (10 μg/mL) and by 100% viability for the solvent control (0.5% DMSO). For the fluorescence microscopy data quantified with CellProfiller, principal component analysis was performed for the 40 parameters of mitochondria. Factors were Kaiser normalized and Varimax rotated. Variables that contributed > 0.85 to the factor loadings of PC1 or PC2 were considered as important. Selected parameters contributing to PC1 or PC2 were tested for normal distribution by the Kolmogorov-Smirnov test. Since data showed a non-parametric distribution, Kruskal-Wallis was applied followed by Dunn´s Multiple Comparison posthoc test. Significant differences were considered if p < 0.05. Data from functional assays (DCFDA, nrf2, alamar blue, ATP) were tested for normality distribution (Kolmogorov-Smirnov) and equal variances (Barthlett´s test). If such criteria were met, One-Way ANOVA was applied followed by Dunnett´s posthoc test; if criteria were not met, Kruskal-Wallis was applied followed by Dunn´s Multiple Comparison posthoc test.

## Supporting information

S1 Fig Relative proportions of portoamides A and B. Absorption spectra (A) obtained by the analytic method with the absorbance as function of time. The first peak represents portoamide A, while the second peak is portoamide B. The PDA spectrum (B), for each absorbance spectrum, with absorbance in the wavelength of 276.0 nm.
(TIF)

S1 Dataset Data from CellProfiler analyses. Raw data are given as derived from the CellProfiler software after analysis of fluorescent images, and included 40 parameters of mitochondria used for the principal component analysis. CS, solvent control; PA, portoamides 0.5 μg/mL; PB, portoamides 1 μg/mL.
(XLSX)
