# Supplementary figures and images for: Cytotoxicity of portoamides in human cancer cells and analysis of the molecular mechanisms of action
Source: PLoS One. 2017 Dec 7;12(12):e0188817. doi: 10.1371/journal.pone.0188817 (PMC5720714; doi:10.1371/journal.pone.0188817)

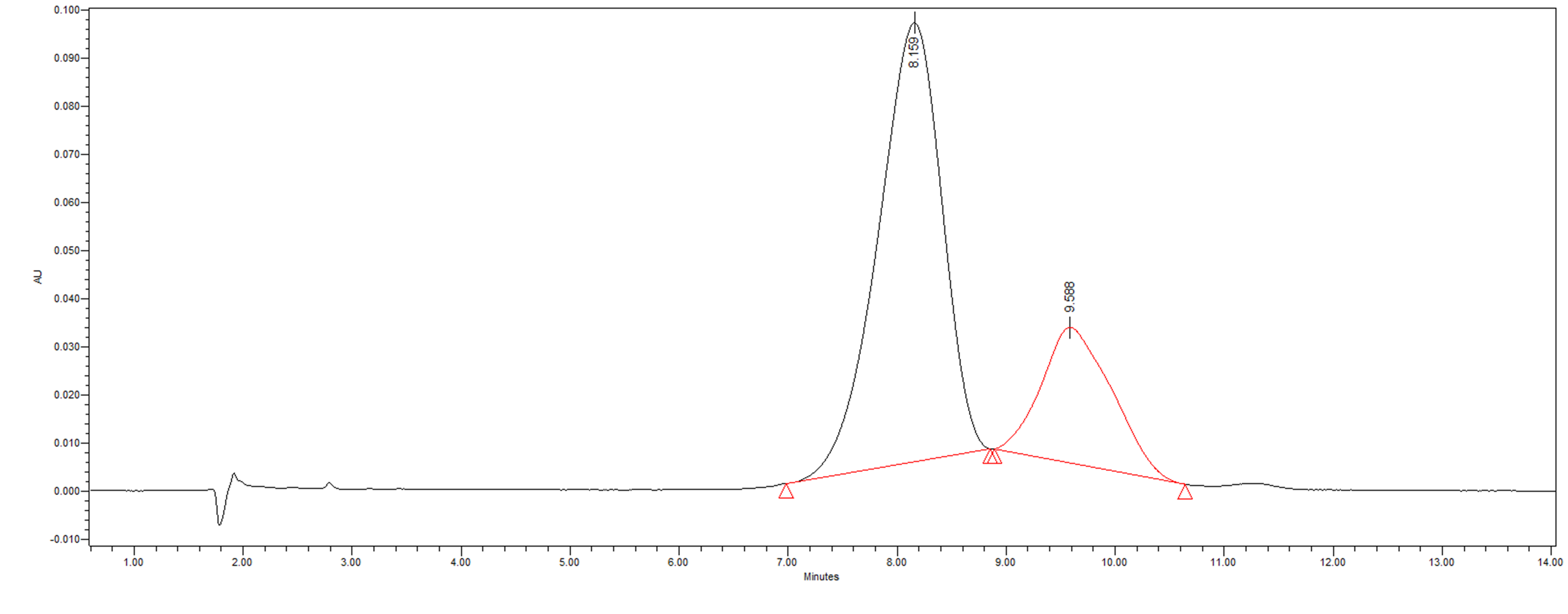

Supplement: S1 Fig — Relative proportions of portoamides A and B. Absorption spectra (A) obtained by the analytic method with the absorbance as function of time. The first peak represents portoamide A, while the second peak is portoamide B. The PDA spectrum (B), for each absorbance spectrum, with absorbance in the wavelength of 276.0 nm. (TIF) [file pone.0188817.s001.tif]
